# Detection of sentinel and non-sentinel lymph node micrometastases by complete serial sectioning and immunohistochemical analysis for gastric cancer

**DOI:** 10.1186/1756-9966-27-7

**Published:** 2008-05-30

**Authors:** Kaname Ishii, Shinichi Kinami, Kenichiro Funaki, Hideto Fujita, Itasu Ninomiya, Sachio Fushida, Takashi Fujimura, Genichi Nishimura, Masato Kayahara

**Affiliations:** 1 Department of Gastroenterologic Surgery, Kanazawa University Hospital, 13-1 Takara-machi, Kanazawa 920-8641, Ishikawa, Japan

## Abstract

**Background:**

We investigated the presence and distribution of the sentinel and the non-sentinel node micrometastases using complete serial sectioning and immunohistochemical staining (IHC), to inspect whether lymph node micrometastases spread to the sentinel lymph nodes first.

**Methods:**

A total of 35 patients, who underwent gastrectomy with a sentinel lymph node biopsy for gastric cancer, were enrolled in this study. Total of 1028 lymph nodes of 35 patients having gastric cancer without metastasis of lymph node by permanent section with hematoxylin and eosin staining (H&E) were selected. There were 252 sentinel nodes and the other 776 were non-sentinel nodes. All nodes were sectioned serially and stained alternately with H&E and IHC. Lymph node micrometastases was defined as proving to be positive first either the IHC or the complete serial sectioning.

**Results:**

Micrometastases were detected in 4 (11%) of the 35 patients, 6 (0.58%) of 1028 nodes. Of these 4 patients, 3 had micrometastases exclusively in sentinel nodes, and the other had micrometastasis in both sentinel and non-sentinel nodes. There was no patient who had the micrometasitases only in non-sentinel nodes.

**Conclusion:**

These results support the concept that lymph node micrometastasis of gastric cancer spreads first to sentinel nodes.

## Background

The prognosis of patients with gastric cancer is influenced primarily by the presence of lymph node metastases. Lymph node metastases in gastric cancer patients with submucosal invasion occur in 15 to 20% of patients; therefore, a lymph node dissection may be unnecessary for the remaining 80 to 85% of patients [1]. An accurate and reliable indicator to predict the absence of lymph node metastases would eliminate many unnecessary lymphadenectomies [1]. Therefore, a preoperative and accurate diagnosis of lymph node metastases remains important [2-4]. A sentinel node biopsy for gastric cancer is an intraoperative diagnostic method to detect lymph node metastases [5-7]. In 1992, Morton et al. [8] introduced the technique of intraoperative dye injection at the site of melanoma to identify the "sentinel" node, which is the first node that the afferent lymphatics enter from the tumor site. Miwa et al. [7,9] employed this type of dye mapping technique to identify the sentinel nodes of gastric cancer, and reported a high positive predictive value and accuracy for the sentinel node biopsy of early gastric cancer. On the other hand, the presence of a micrometastasis in a lymph node is a serious issue for the clinical application of sentinel node biopsy for early gastric cancer. Lymph node micrometastases have been found in patients determined to be node-negative by routine histological examination. Previous investigators have reported that lymph node micrometastases could be detected using step sectioning, immunohistochemical staining and the reverse transcriptase-polymerase chain reaction [10-12]. However, there have been a few reports about the distribution of micrometastases in both the sentinel and non-sentinel nodes in node-negative gastric cancer patients by routine histologic examination [13-15].

In this study, we retrospectively investigated the presence and distribution of sentinel and non-sentinel node micrometastases using complete serial sectioning and immunohistochemical staining. These technique are the most accurate methods to detect micrometastases in nodes so that we could determine whether lymph node micrometastases had spread to the sentinel lymph nodes first.

## Methods

A sentinel lymph node (SLNs) biopsy for gastric cancer was performed on 243 patients at the Department of Gastroenterologic Surgery, Kanazawa University Hospital from 1993 to 2002. Before the sentinel node biopsy was performed, written informed consent was obtained in accordance with the ethical standards of the Committee on Human Experimentation of Kanazawa University Hospital. Of these patients, we enrolled 35 who had a cancer that had invaded to the submucosa or muscularis propria and had no lymph node metastasis by routine histologic examination for this study. None of the patients had received preoperative chemotherapy or radiotherapy. Based on the Japanese Classification of Gastric Carcinoma, all 35 patients underwent a sentinel node biopsy followed by conventional lymphadenectomy for back-up dissection [16]. A total of 1028 lymph nodes were removed from the 35 patients. Of these, 252 lymph nodes were SLNs and the other 776 were non-SLNs. All 252 SLNs were negative for metastases both on intraoperative frozen-section examination and permanent section with hematoxylin and eosin staining (H&E). The other 776 nodes were negative for metastases on histological examination by H&E of multiple step sectioning at 0.2 cm intervals. The clinicopathologic data were evaluated according to the Japanese Classification of Gastric Cancer [16]. The patients characteristics are listed in Table 1. For detecting the SLNs, we used intraoperative endoscopic lymphatic mapping (IELM), which consisted of an intraoperative injection of 0.2 ml of 2% patent blue into the submucosal layer at four sites around the gastric carcinoma through a gastroscope [9]. The dye immediately appeared at the serosal surface and stained the lymphatic vessels and nodes [5,9]. In this study, the SLN was defined as the lymph node that stained blue 20 minutes after the injection. The lymphatic basins were defined as the area containing the stained lymphatic vessels, and which were able to be divided into five categories according to the directions of the arteries surrounded the stomach, as follows: the left gastric artery area, the right gastric artery area, the right gastroepiploic artery area, the right gastroepiploic artery area and the posterior gastric artery area. The excised SLNs were sent for frozen-section examination. The lymph nodes stained with H&E on representative sections were cut along the plane with the largest diameter that included the node hilus, and examined intraoperatively for metastases.

**Table 1:** Patients characteristics

Median age (range)	62 (37–85)
Sex	
Male	23
Female	12
Depth of invasion	
SM	24
MP	11
Histological type	
Differentiated *	18
Undifferentiated * *	17
Lymphatics invasion	
Negative	16
positive	19
Vascular invasion	
Negative	30
positive	5

SM, submucosa; MP, muscularis propuria

*The differentiated type;papillary, well and moderately differentiated tubular adenocarcinomas

**The undifferentiated type;poorly differentiated adenocarcinoma, mucinous adenocarcinoma and signet-ring cell carcinoma.

The remaining frozen tissues were thawed, and the tissues and non-SLNs were routinely cut at 0.2 cm intervals. Subsequently, the multiple sectioned lymph nodes and resected specimens were fixed in 10% formalin, processed through graded ethanol, and embedded in paraffin for permanent sections. The lymph nodes were stained with H&E and were examined by two pathologists.

In this study, all resected lymph nodes were sectioned serially at 25-μm intervals of 4-μm thickness in addition and either alternately stained with H&E and immunohistochemical staining (IHC) using an anti-cytokeratin antibody. The ENVISION technique was used (DAKO, Carpinteria, CA) for IHC and we used the monoclonal anti-human cytokeratin 8/18 antibody (Santa Cruz Biotechonology, California, USA) [17-19]. All main tumor specimens from 35 patients were subjected to cytokeratin staining and were used as a positive control. A lymph node micrometastasis was defined as a node negative for metastasis by our routine histologic examination, but positive by either the IHC or the complete serial sectioning methods.

## Results

**Figure 1:**
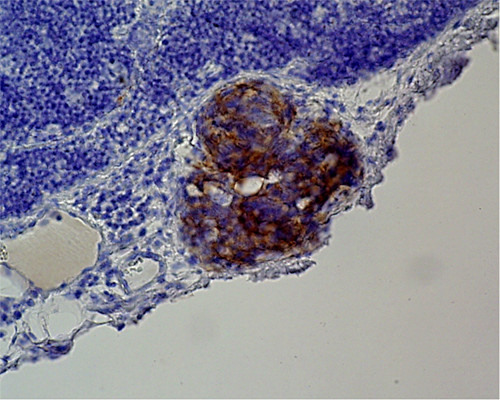
Lymph node mircrometastasis as detected by immunohistochemical staining with a cytokeratin antibody. (×400).

**Figure 2:**
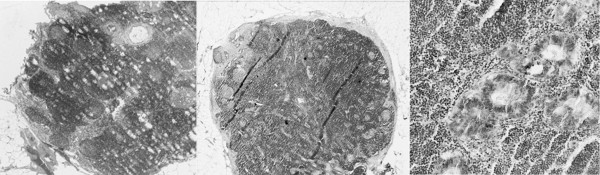
No cancer cells were identified in this section by routine histologic examination. (hematoxylin & eosin staining, ×40). (b) Lymph node micrometastasis in the representative section by the entire serial sectioning method. (Immunohistochemical staining, ×40). (c) Micrometastasis lymph node in the representative section by the entire serial sectioning method. (Immunohistochemical staining, ×400)

The total number of sections examined was 24,094. Of these, 5986 were SLNs and 18,108 were non-SLNs. Of the 35 patients, 4 (11%) had micrometastases. A micrometastasis was found in 6 of 1028 nodes (0.6%) and 60 sections (0.3%) of 24,094 (Figs. 1, 2). Of these 6 nodes involving a micrometastasis, 4 were SLNs and the other 2 were non-SLNs in the lymphatic basin. No micrometastases were detected outside the basin (Table 2). The details of the distribution pattern, location and size of the micrometastases are shown in Tables 3, 4 and 5. Of the 4 patients who had a lymph node micrometastasis, 3 patients had micrometastases exclusively in the SLNs. The other patient had a micrometastasis in both the SLN and non-SLNs in the lymphatic basin. No patient had a micrometastasis only in non-SLNs. No patient has yet suffered a recurrence or has died as of December, 2007.

**Table 2:** Number and location of lymph nodes with micrometastasis

Location of nodes	Number of nodes	
	
	with micrometastasis	without micrometastasis
SLNs	4	248
non-SLNs in lymphatic basin	2	653
non-SLNs out of basin	0	121

All nodes	6	1022

SLNs, sentinel lymph nodes

**Table 3:** Distribution pattern of lymph node micrometastasis

	Number of cases	
	
Distribution pattern of micrometastasis	Routine histological examination	complete serial sectioning and IHC
SLNs (-), non-SLNs (-)	35	31
SLNs (+), non-SLNs (-)	0	3
SLNs (+), non-SLNs (+)	0	1
SLNs (-), non-SLNs (+)	0	0

SLNs, sentinel lymph nodes; IHC, immunohistochemic staining

**Table 4:** Location of lymph node micrometastasis

case	Location of tumor	Stained lymphatic basins	Number of SLNs	Number of micrometastsis	Station of micrometastasis of SLNs	Station of micrometastasis of non-SLNs
1	M, Less	Left GA	8	1	No.3 LN	-
2	M, Post.	Left GA Right GEA	5	1	No.3 LN	-
3	M, Ant.	Left GA	2	1	No.3 LN	-
4	M, Less	Left GA Right GEA	10	3	No.3 LN	No.3 LN

SLNs, sentinel lymph nodes; M, Middle third of the stomach; Less, lesser curvature; Post.; posterior wall; Ant. anterior wall; GA, gastric artery; GEA, gastroepiploic artery; No.3 LN, LN along the lesser curvature

**Table 5:** Site and size of lymph node micrometastasis

case	Type of lymph node	Site in lymph node	Size of micrometastasis(mm)
1	SLN	peripheral sinus	1.2
2	SLN	peripheral sinus	0.3
3	SLN	peripheral sinus	0.2
4	SLN	peripheral sinus	0.6
	non-SLN	peripheral sinus	1.0
	non-SLN	peripheral sinus	1.0

SLN, sentinel lymph node

## Discussion

Miwa et al. introduced the concept of sentinel node biopsy for gastric cancer [7]. The clinical use of the sentinel node biopsy to determine the surgical approach for gastric cancer requires the verification of this concept at the level of lymph node micrometastases. In this study, we investigated the presence and distribution of lymph node micrometastases in patients with gastric cancer who had a sentinel node biopsy. Recently, the presence of lymph node micrometastases undetectable by routine histological examination has been reported in breast, lung, esophagus, stomach, colon and gallbladder cancers. It has been reported that a lymph node micrometastasis was a poor prognostic indicator in breast, lung, and colon cancers [20-22]. A few authors have reported that a lymph node micrometastasis was a poor prognostic factor in gastric cancer patients [23,24]. Thus, the importance of the detecting a lymph node micrometastasis has been emphasized for various neoplastic diseases.

A variety of methods to detect lymph node micrometastases exist, including IHC and polymerase chain reaction assays. Matsumoto et al. demonstrated that the reverse transcriptase-polymerase chain reaction (RT-PCR) is more sensitive than IHC for the detection of micrometastases [2]. However, Yamamoto et al. [25] suggested that positive results with a molecular assay such as RT-PCR may not be indicative of the presence of viable tumor cells, but rather the presence of tumor DNA and thus, may be associated with a greatly increased false positive rate despite the higher sensitivity of the molecular assay. On the other hand, it has been reported that the serially sectioning increased the identification of tumor cells in the peripheral sinuses of lymph nodes [26]. It is thought that serial sectioning with IHC is the most accurate method for the detection of lymph node micrometastases. Therefore, we subjected the entire specimen to serial sectioning and IHC. The antibody used for IHC was a monoclonal anti-human cytokeratin 8/18 antibody which is more sensitive, specific, simple, accurate, and economic than other antibodies for IHC.

Tumor deposits within lymph nodes were classified and staged according to the revised guidelines set by the International Union Against Cancer (UICC) 6^th ^Edition. According to this classification system, metastases less than 0.2 cm were considered micrometastases (MMs), and isolated tumor cells (ITCs) were single tumor cells or small clusters of cells that measured no greater than 0.2 mm and were usually detected by IHC or molecular methods, but may be verified with H&E. ITCs do not typically show evidence of metastatic activity by proliferation, induction of a stromal reaction, or penetration of vascular or lymphatic sinus wall invasion [27,28]. Nakajo et al. [23] and Siewert et al. [29] reported that lymph node involvement is classified into cluster formation or single cell forms, according to the results of IHC for cytokeratin. Their results suggested that single cells cannot proliferate in lymph nodes because they were already killed by local and general immunocytes. A cluster of cells with a stromal reaction may easily proliferate and therefore have metastatic potential. In our department, resected lymph nodes are routinely cut at 0.2 cm intervals and the lymph nodes are examined for metastases. So, in this study, the definition of lymph node micrometastasis differed from the UICC classification. For this study it was defined as a node, negative for metastasis by our routine histological examination of sections cut at 0.2 cm intervals, but positive by complete serial sectioning with H&E and IHC. In addition, all single cell types and small cluster types without a stromal reaction by cytokeratin positive staining were not recognized as cancer cells in the next H&E stained slide. Thus, we excluded all single cells and small clusters without a stromal reaction, which are classified as ITCs in the UICC classification system, from the positive lymph node micrometastasis group [27,28].

We excluded the gastric cancer patients whose tumors had invaded to the mucosa in this study because doing complete serial sectioning and immunohistochemical staining was a lot of work; in addition, the rate of a lymph node micrometastasis was low. Accordingly we enrolled 35 who had a gastric cancer that had invaded to the submucosa or muscularis propria and had no evidence of a lymph node metastasis by histologic examination for this study.

In this study, we observed a lymph node micrometastasis in 4 patients (11%) with a gastric cancer that had invaded to the submucosa or muscularis propria. In gastric cancer, Isozaki et al. [30] and Natsugoe et al. [31] reported that lymph node micrometastases were identified in 10 to 30% of specimens by step-sectioning or IHC. Our results were the most accurate of all the past studies and proved the actual circumstances of lymph node micrometastasis of gastric cancers that had invaded to the submucosa or muscularis propria.

In this study, we examined the lymph node micrometastases of SLNs and non-SLNs. We found lymph node micrometastases in the SLNs of 4 patients. One patient also had a micrometastasis in a non-SLN of the lymphatic basin, though no micrometastases of non-SLNs were identified outside the basin. Furthermore, no patient had a lymph node micrometastasis only in a non-SLN. Our results revealed that the patients who didn't have a lymph node micrometastasis in the SLNs also didn't have a micrometastasis in the non-SLNs. These results may support the concept that lymph node micrometastases spread first to the SLNs, then to the non-SLNs in the lymphatic basin and finally to non-SLNs outside the basin. Therefore, based upon this concept, it is sufficient to examine only the SLNs to determine whether or not there are lymph node micrometastases in patients with gastric cancer.

It is still unclear whether a lymph node micrometastasis is a prognostic factor in gastric cancer. However, a lymph node micrometastasis was found in gastric cancer patients who had no evidence of a lymph node metastasis by routine staining. This result suggests that we should cautiously reduce the extent of lymph node dissections. The intraoperative absence of a SLN micrometastasis suggests that the extent of lymph node dissection may be safely reduced, because it is unlikely for non-SLNs to have micrometastases without a SLN micrometastasis. In the case of breast cancer, the need for the intraoperative diagnosis of lymph node micrometastases is not essential, because additional dissection of the axillary lymph nodes can be performed easily. However, the subsequent dissection of lymph nodes is difficult in gastric cancer; therefore, the intraoperative diagnosis of lymph node micrometastases is crucial. We believed that when a lymph node micrometastasis was present, we should perform a lymph node dissection at the present. Our study utilized the most accurate methods, but we could not obtain the results rapidly enough for an itraoperative diagnosis. Therefore, we need to establish an accurate method for rapid intraoperative identification. Matsumoto et al. claimed that intraoperative rapid immunostaining was a simple and useful technique for detecting lymph node micrometastases [32]. An ultra-rapid RT-PCR system, which can complete the detection of cancer cells within approximately 70 minutes, has been developed. In the near future, these methods will be applied to detect lymph node micrometastases in SLNs during surgery [33].

## Conclusion

we have demonstrated the ability to detect lymph node micrometastases by subjecting the entire specimen to complete serial sectioning and IHC for node-negative gastric cancer patients who have had a sentinel node biopsy. These results support the concept that lymph node micrometastases spreads first to the SLNs. In addition, the intraoperative and rapid diagnosis of lymph node micrometastases in SLNs may help guide the appropriate lymph node dissection in gastric cancer patients. Therefore, a rapid and accurate intraoperative diagnosis of lymph node micrometastases in SLNs will be necessary and should be the focus of future studies.
